# Employees and the National Insurance Act

**Published:** 1912-08-24

**Authors:** 


### NATIONAL INSURANCE ACT DEPARTMENT.

### EMPLOYEES AND THE NATIONAL INSURANCE ACT.

### XXV.—Existing Funds of Friendly Societies.

The arrangements made in the Act with respect to the existing funds of Friendly Societies are contained in Section 72, and as the subject is closely allied to that with which we dealt last week—namely, the financial provisions—we propose to consider it in some detail. It will be remembered that by taking into insurance a very large number of persons over the age of sixteen years reserves have to be set up for reasons we explained last week. These reserves are not merely to be dealt with in bulk, but an individual account is to be taken and the proper allowances depending upon the age at entry into insurance, and, in the case of women, upon their condition (whether married or spinster or widow), are to be credited to the approved societies. Thus every approved society will obtain the necessary credit to meet the deficiency which would otherwise have arisen by its acceptance of members over the age of sixteen at the ordinary rates of contribution. We have already explained the advantages of this procedure to those who become members of approved societies, for it is only on account of such persons that the reserves are set up; those who become deposit contributors in the Post Office Fund, since they have no real insurance, have no claim on reserve values.

Special Advantages for Existing Members.

Apart from these advantages and those which attach generally to membership of an approved society there is a special advantage which applies to those who were members of Friendly Societies before the passing of the Act—that is, before December 16, 1911. The advantage arises in this way. If the society was constituted on the sound "accumulation plan" it would have in hand on account of each member a certain reserve to provide for future liabilities in respect of that member on the basis of the benefits guaranteed. In so far as those benefits are the same as the benefits provided under the Act, that reserve which the society has itself accumulated is no longer required if the member decides to lapse the whole or a part of his existing insurance, taking instead the compulsory State insurance. Thus it is laid down in Section 72, sub-section (1), of the Act that:

Every registered Friendly Society, which provides benefits similar to any of those conferred by the Act, shall submit to the Registrar of Friendly Societies a scheme for continuing, abolishing, reducing or altering such benefits as respects members who become insured persons, and for continuing, abolishing or reducing the contributions of such members, so, however, that the combined effect of the alteration of the benefits and contributions shall not prejudicially affect the solvency of the society, and, if the scheme or a supplementary scheme shows on an actuarial valuation that, owing to the alterations in the benefits and contributions effected by the scheme, any part of the existing funds of the society is set free as not being required to meet the liabilities of the society, the scheme or the supplementary scheme shall provide for the application of the part of the funds so set free in any one or more of the following ways: (*a*) towards the cost of the provision of other or increased benefits payable by the society independently of the Act to existing members, whether insured persons or not; (*b*) in reduction of the contributions payable by such members in respect of the benefits payable by the society independently of the Act; and (*c*) towards the payment or repayment of contributions payable under the Act by such of its existing members as are entitled and elect to receive benefits under the Act through the society.

We have quoted this long sub-section in full because it expresses in a very clear and precise manner exactly what is to happen, but we can well imagine that its full significance may not at once be appreciated. It should be noted in the first place that the provision applies to *every* registered Friendly Society, and not only to societies that become approved for the purposes of the Act, and thus every person insured under the National scheme, who at the time of the passing of the Act was a member of a registered Friendly Society, is entitled to benefit under the scheme that must be drawn up by his society, whether it becomes approved or not. It is, of course, open to a society to submit a scheme for continuing the contributions and benefits as before, but such a scheme will in many cases work a hardship to those who are not in a financial position to pay their State contributions and their existing contributions to their society as well. A scheme may also give an option to members who become insured persons either to continue their present contributions and benefits or to pay contributions and receive benefits at a reduced rate, but it may not give such an option only to those members who elect to take the society as their approved society, compelling the other members who become insured persons and do not take that society as their approved society to continue their contributions and benefits as formerly. The sub-section we have quoted does not allow of such discrimination.

The ideal would, of course, be to allow existing members the option of reducing their private insurances, if they wish to do so, by making a reduction in their contributions equivalent to 4d. a week in the case of men and 3d. a week in the case of women, as these are the amounts that have to be paid weekly by the insured person under the Act. It is not possible, however, for this reduction to be made in all cases, because the scheme for alteration must not prejudicially affect the solvency of the society, and, further, that the benefits conferred by the Act very seldom, if ever, exactly coincide with those granted by any particular society. There is, for instance, the departure from Friendly Society practice on the sickness benefit conferred by the Act that it only becomes payable as from the fourth day of sickness, instead of from the first day. Again, there are the waiting periods imposed by the Act before benefits begin to accrue, whilst on its private side a society would not be relieved of its liability to pay claims arising during such periods if the member continues his contributions. Furthermore, it has to be remembered that under the Act contributions do not have to be paid for the period when the insured person is in receipt of sickness or disablement benefit, whilst it is the usual practice of Friendly Societies to require contributions to be paid whether the member is well or ill.

### The Difficulty of Drafting a Scheme.

It will at once be seen that the drawing up of a suitable scheme is a matter of considerable complexity, and although Section 72 came into operation on December 16, 1911—that is, on the passing of the Act—very few societies have as yet completed their arrangements. There was the added difficulty of determining in advance of the commencement of the Act (July 15, 1912) which members of the societies would come within its operation, and, in fact, this matter cannot even yet be definitely settled in all cases. Many Friendly Societies have amongst their members those whose remuneration from their employers exceeds the limit of £160 a year, and who will in consequence not be entitled to benefit under the Act, unless they are engaged in manual labour. A large number of members of Friendly Societies would also come under the description of those who are eligible for insurance under the Act as voluntary contributors; thus there cannot be any finality in any scheme or supplementary scheme that may be adopted until such time as these members have determined whether to exercise their option of entering the State insurance or not. In order to meet this condition of affairs the Registrar of Friendly Societies issued some time back a circular setting forth suggestions for an appropriate scheme, and to meet the difficulty of obtaining confirmation of a permanent scheme before July 15 the Registrar was prepared to consider, with a view to confirmation, a provisional scheme for the reduction of members' contributions until the permanent scheme is confirmed, provided that such provisional scheme did not appear to prejudicially affect the solvency of the society submitting it. A table was furnished with this circular from which a rough idea of the amount of deduction that could be made from the contributions might be determined. This table showed in the left-hand column various degrees of solvency of societies, and to make use of the table it was necessary to ascertain the degree of solvency of the society from its last valuation report. The table then proceeded to show in columns headed A to M, with various rates of sickness benefits in force in societies, the amounts of reduction of contribution appropriate to the degree of solvency as shown in the left-hand column. The deductions also vary according as medical attendance is provided by the society or not.

The circular points out that as a general rule it will probably be found in the average society paying the sickness and medical benefits provided by the Act, that if the benefits are modified to correspond with those under the Act in the matter of detail, and the contributions are reduced by 4d. a week, the solvency of the society will not be injured if its worth on a sound actuarial valuation exceeds 15s. in the £. Smaller reductions of contributions will, however, be necessary if the society provides much lower benefits than those named in the Act and is in serious deficiency, and the same result may follow if the members are not distributed as to age in similar proportion to an average society. In cases like this the member will have to pay 4d., it is true, to the State insurance, and thus may have to pay more in total than formerly; but, on the other hand, he will get larger benefits, with greatly enhanced security for their due payment. The determination of the amount of reduction is, of course, a matter for actuarial advice.

### Use of the Funds set Free.

Whilst under the scheme or provisional scheme it may not be possible to reduce the contribution by the amount of that required for the State insurance it may be feasible to effect the reduction under the supplementary scheme when disposing of the funds set free as not being required to meet the liabilities of the society. There is no necessity for the alteration of benefits and contributions to be made in the first instance in such a way as to increase the degree of solvency of the society; this may happen, of course, but the only condition prescribed is that the financial position of the society shall be no worse after the alteration than it was before it. The portion of the existing funds set free must apparently in the first instance be used towards making the society solvent, for the words employed are "as not being required to meet the *liabilities of the society*," that is, presumably, of the society as a whole and not merely on account of those affected by the Act. The use to which the surplus may be put as described in clause (*a*) clearly indicates the intention of the Act to benefit those members of Friendly Societies who do not become insured, as well as those who do, for the words are "to existing members whether insured persons or not." The method (*c*) can only be adopted when the society becomes approved and the member is entitled and elects to receive his State benefits through his society. The schemes and supplementary schemes with which we have been dealing must be drawn up for each branch of a society with branches, unless it is possible to submit a scheme applicable to all its branches.

There is one further point: that the special advantages with which we have been dealing can only apply to those who were existing members of Friendly Societies on the passing of the Act. No Friendly Society that becomes approved can offer immediately any better benefits to new members than any newly formed approved society, and it is doubtful if they will ever be able to do so. Those who have for years contributed to a well-conducted Friendly Society are acting in their own best interests in taking their State insurance through their society if it becomes approved, but the special benefits they enjoy are really the result of their own thrift, and do not extend to those who have not exercised it.

